# Definitions of hospital-acquired pneumonia in trauma research: a systematic review

**DOI:** 10.1007/s00068-024-02509-8

**Published:** 2024-03-28

**Authors:** Tim Kobes, Diederik P. J. Smeeing, Falco Hietbrink, Kim E. M. Benders, R. Marijn Houwert, Mark P. C. M. van Baal

**Affiliations:** 1 Department of Trauma Surgery, University Medical Center Utrecht, PO Box 85500, 3508GA Utrecht, The Netherlands; 2 Department of Surgery, Rijnstate Hospital, Arnhem, The Netherlands; 3 Department of Trauma Surgery, St Antonius Hospital, Utrecht, The Netherlands

**Keywords:** Hospital-acquired pneumonia, Trauma patient research, Clinical definition, Guideline criteria, Diagnostic criteria, Diagnosis

## Abstract

**Purpose:**

What are reported definitions of HAP in trauma patient research?

**Methods:**

A systematic review was performed using the PubMed/MEDLINE database. We included all English, Dutch, and German original research papers in adult trauma patients reporting diagnostic criteria for hospital-acquired pneumonia diagnosis. The risk of bias was assessed using the MINORS criteria.

**Results:**

Forty-six out of 5749 non-duplicate studies were included. Forty-seven unique criteria were reported and divided into five categories: clinical, laboratory, microbiological, radiologic, and miscellaneous. Eighteen studies used 33 unique guideline criteria; 28 studies used 36 unique non-guideline criteria.

**Conclusion:**

Clinical criteria for diagnosing HAP—both guideline and non-guideline—are widespread with no clear consensus, leading to restrictions in adequately comparing the available literature on HAP in trauma patients. Studies should at least report how a diagnosis was made, but preferably, they would use pre-defined guideline criteria for pneumonia diagnosis in a research setting. Ideally, one internationally accepted set of criteria is used to diagnose hospital-acquired pneumonia.

**Level of evidence:**

Level III.

**Supplementary Information:**

The online version contains supplementary material available at 10.1007/s00068-024-02509-8.

## Background

Nosocomial pneumonia is among the most frequent complications in trauma patients and is associated with increased mortality and poor prognosis [1–3]. The incidence of nosocomial pneumonia ranges from 4.3 to 38.3% in the literature, and this wide variety may cast doubt on the individual studies’ comparability [4, 5].

Several types of nosocomial pneumonia have been described in the literature [6]. Most guidelines on nosocomial pneumonia create a distinction between hospital-acquired pneumonia (HAP) and ventilator-associated pneumonia (VAP) [7–9]. Although VAP essentially is a particular type of HAP, the etiology is not the same. In VAP, endotracheal intubation enables upper respiratory tract colonization by inserting a foreign body; therefore, the two pneumonia types should not be considered equivalent [10]. Nonetheless, the diagnostic criteria are similar for HAP and VAP in most guidelines, though they differ in the exact duration of mechanical ventilation and the time between mechanical ventilation and pneumonia onset to distinguish VAP from HAP [7–9].

To diagnose hospital-acquired pneumonia, microbiologic diagnostics are superior to clinical symptoms or radiologic examination [10]. Collecting sputum or tracheal secretions has high sensitivity but low specificity, while bronchoalveolar lavage and comparable methods have both high sensitivity and specificity. However, as fluid is introduced into the lungs, bronchoalveolar lavage is generally unsuitable for non-mechanically ventilated patients and is, therefore, mainly used to diagnose VAP [11]. Thus, HAP diagnosis is reliant on clinical criteria.

The combination of varying incidence and diagnostic criteria reliance raises the question of what criteria have been previously used to diagnose HAP in trauma patient research [12, 13]. Potentially, HAP incidence varies because of the use of different diagnostic criteria. Therefore, this systematic review was conducted to create an overview of reported definitions of hospital-acquired pneumonia in trauma research.

## Methods

This systematic review was performed according to the Preferred Reporting Items for Systematic research and Meta-Analysis (PRISMA) checklist and registered on PROSPERO (review identification number CRD42022350131) [14].

### Search strategy and execution

A literature search was performed in PubMed/MEDLINE. The search syntax was constructed to identify studies that stated a definition for pneumonia (Supplemental Table 1) from initiation to September 2019. The search syntax included the following: the MeSH terms and subheadings “Wounds and Injuries,” “Injuries,” “Pneumonia,” “Incidence,” “Prevalence,” “Risk Factors,” and “Prevention and Control”; keywords derived from the MeSH terms and subheadings; and additional keywords on trauma patients, clinical criteria, definitions, prediction, and prophylaxis. Animal studies were excluded from the syntax.

### Review process

The search results were imported into Rayyan for processing [15]. Rayyan is a free online tool that helps researchers conduct systematic reviews. Studies in trauma patients with a reported definition of HAP were included, with no limitations set on the type of trauma. We excluded certain study populations (pediatric, burns, (near-)drowning, non-traumatic fractures, postmortem), other entities of pneumonia or pulmonary complications (solely as an outcome or mixed with HAP), non-original research papers, and studies in a language other than English, Dutch, or German. We assumed that all Intensive Care Unit admitted patients were at risk for VAP unless stated differently. Subsequently, we excluded studies that did not use clinical criteria to diagnose HAP but presented references to these studies separately in Supplemental Table 2.

One reviewer (TK) assessed the in- and exclusion stepwise: first, the patient population; second, the pneumonia outcome; and lastly, other remaining criteria. The same reviewer assessed the methodological quality using the MINORS criteria: a clarification of used criteria can be found in Supplemental Table 3 [16]. The possible score on the MINORS criteria ranges from 0 (lowest) to 24 (highest) for comparative studies. In non-comparative studies, 16 is the highest possible score. Any borderline cases were discussed with a second reviewer (DS) before definitive in-/exclusion or quality scoring.

For each study, the following data were obtained: first author, year of publication, study period, study design, cohort size, and the applied diagnostic criteria. All data extraction was conducted by one reviewer (TK).

## Results

**Fig. 1:**
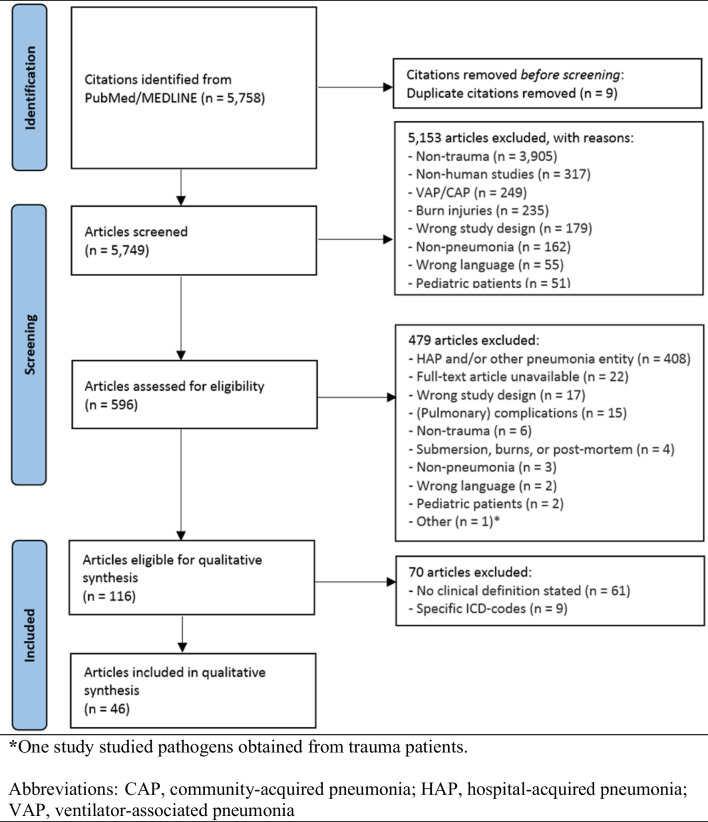
The PRISMA flow diagram, illustrating the in- and exclusion process of studies on trauma patients with a reporting, clinical definition of HAP

The PubMed/MEDLINE database search resulted in 5758 studies. One hundred and sixteen studies were eligible for the qualitative comparison; seventy studies (60%) did not use clinical criteria to diagnose HAP (e.g., medical records or ICD-codes; Supplemental Table 2). The remaining 46 studies were included in the qualitative analysis [12, 17–64]. The study selection process is summarized in the PRISMA flowchart (Fig. 1). The included studies were performed retrospectively (21/46) and prospectively (25/46). Table 1 shows the baseline characteristics of the included studies.

**Table 1:** Baseline characteristics of included studies

Author	Year	Country	Study period	Design	Cohort size	Study quality^§^
Seok [19]	2019	Korea	2013–2018	Retrospective, observational, single-center study	207	14
Conradsson [20]	2019	South Africa	2013–2014	Prospective, population-based cohort study	139	14
Warren [18]	2019	United States	2014–2016	Quasi-experimental pretest–posttest evaluation plan	417	12
Wutzler [17]	2019	Germany	2010–2014	Retrospective, observational study	1,162	15
Djuric [23]	2018	Serbia	2014–2016	Prospective patient-based, single-center surveillance study	406	20
Guo [22]	2018	China	2010–2016	Randomized double‐blind, placebo‐controlled clinical trial	204	15
Yadollahi [21]	2018	Iran	2015–2017	Prospective cohort	10,553	11
Denis [24]	2018	Canada	2010–2015	Prospective cohort study	159	16
Folbert [26]	2017	The Netherlands	2011–2013	Naturalistic cohort study	452	14
Yoo [25]	2017	Korea	2010–2014	Prospectively compiled database was used to identify retrospective patients	272	17
Curtis [27]	2016	Australia	2014	Retrospective before-after cohort study	546	21
Ewan [61]	2015	England	2009–2010	Prospective study	90	9*
Yun [28]	2015	United States	2009–2010	Multicenter, observational cohort	423	9*
Kamiya [29]	2015	Japan	2009–2012	Retrospective comparative analysis using an historical cohort control	62	14
Landeen [30]	2014	United States	2005–2011	Retrospective observational study	364	18
Yang [12]	2014	United States	2003–2011	Single-center retrospective cohort study	619	15
Mica [32]	2013	Switzerland	1996–2007	Retrospective study	628	16
Hyllienmark [33]	2013	Sweden	2007–2011	Retrospective cohort study	322	12*
Schirmer-Mikalsen [31]	2013	Norway	2004–2009	Prospective study	133	14
Yeung [34]	2012	United States	2003–2010	Patient control study	162	22
Hakim [35]	2012	Egypt	2008–2011	Randomized, parallel-arm, open-label study	55	7*
Strumwasser [36]	2011	United States	2005–2010	Retrospective study	106	7*
Becher [23]	2011	United States	2008–2009	Retrospective study	116	12
Karunakar [24]	2010	United States	1997–2005	Retrospective review	110	16
Worrall [25]	2010	United States	*Unknown period*	Retrospective analysis	130	10
García-Alvarez [26]	2010	Spain	1998–2001	Prospective study	290	11
Friese [27]	2008	United States	2000–2003	Retrospective, observational cohort analysis	678	13
Schirmer-Mikalsen [28]	2007	Norway	1998–2002	Retrospective study	133	11
Giamberardino [29]	2007	Brazil	2000–2001	Retrospective study	416	9
Bochicchio [31]	2004	United States	1997–1999	Prospective study	182	11
Kamel [32]	2003	United States	1997–1999	Retrospective observational study	131	14
McKinley [33]	2002	United States	*Unknown*	2-year prospective data comparison	117	12
Carson [34]	1999	United States	1983–1993	Retrospective cohort study	9,598	12
Claxton [35]	1998	Canada	1981–1994	Retrospective study	72	14
Bozorgzadeh [36]	1999	United States	*Unknown period*	Prospective, randomized study	300	15
Gonzalez [37]	1998	United States	1992–1995	Double-blind randomized clinical trial	139	15
Allen [38]	1997	United States	*Unknown 4-year period*	Retrospective review	210	8
Morrison [39]	1996	United States	1989–1994	Retrospective cohort study	80	6*
Renz [40]	1995	United States	1988–1991	Prospective case series	254	8*
Nichols [41]	1994	United States	1988–1992	Double-blind, randomized clinical trial	119	19
Beraldo [42]	1993	Brazil	1989	Review study	664	4*
Rello [43]	1992	Spain	1988–1990	Prospective Study	161	14
Moore [44]	1989	United States	1984–1987	Prospective, randomized study	308	15
Moore [45]	1989	United States	1985–1987	Prospective, randomized study	59	13
LoCurto [46]	1986	United States	1984–1985	Prospective, randomized study	58	13
Grover [47]	1977	United States	*Unknown*	Double-blind prospective study	75	13

^§^The study quality was measured using the MINORS criteria; the potential score ranges from 0 (lowest) to 16 or 24 (highest)

^*^These studies could score a maximum of 16 points

### Diagnostic criteria

Forty-eight unique criteria were described in the included studies. We divided the criteria into five main categories: clinical (pulmonary symptoms and vital signs), laboratory (e.g., C-reactive protein, leukocytes), microbiologic (cultures or pathology), radiologic (X-ray or computed tomography), and miscellaneous (prescribed antibiotics and diagnosis in the medical health record). Radiologic criteria were most commonly used in the included studies (45/46) [12, 18–63]. Clinical, laboratory, and microbiologic criteria were applied in 72, 28, and 39 percent of the included studies, respectively. Miscellaneous criteria were present in eight studies: four studies with only non-guideline criteria [12, 28, 30, 61] and as an addition to guideline criteria in the other four other studies [26, 27, 45, 48].

Guideline criteria were used to diagnose HAP in 18 out of 46 studies (Table 2). The five guidelines that were used originated from the United States of America or Europe: the Centers for Disease Control and Prevention (CDC), the European Center for Disease Prevention and Control (ECDC), the American Thoracic Society/Infectious Disease Society of America (ATS/IDSA), the Swedish Intensive Care Registry (SIR), and the British Society for Antimicrobial Chemotherapy (BSAC). The CDC criteria were cited in 13 out of 18 studies, whereas the ATS/IDSA, ECDC, SIR, and BSAC guidelines were used in the remaining four studies. Two studies applied the criteria of two different guidelines: Djuric et al. used the CDC and ECDC guidelines, and Ewan et al. used the ATS and BSAC guidelines [23, 61]. In the studies that used guideline criteria, 33 unique criteria were observed. The remaining 28 out of 46 studies described 37 non-guideline criteria to diagnose HAP (Table 3).

**Table 2:**
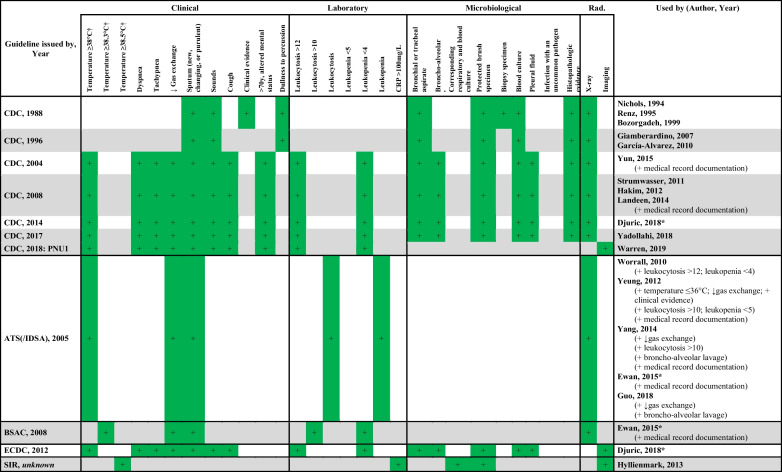
Pre-defined guideline criteria used to diagnose hospital-acquired pneumonia in trauma patient research

†: ≤ and < , and ≥ and > are used interchangeably in this table when body temperatures are corresponding between studies

^*^These studies used more than one guideline

Abbreviations: ATS, American Thoracic Society; BSAC, British Center for Antimicrobial Chemotherapy; CDC, Centers for Disease Control and Prevention; CRP, C-reactive protein; ECDC, European Center for Disease Prevention and Control; Rad., radiological; SIR, Swedish Intensive Care Registry; ↓, worsening

**Table 3:**
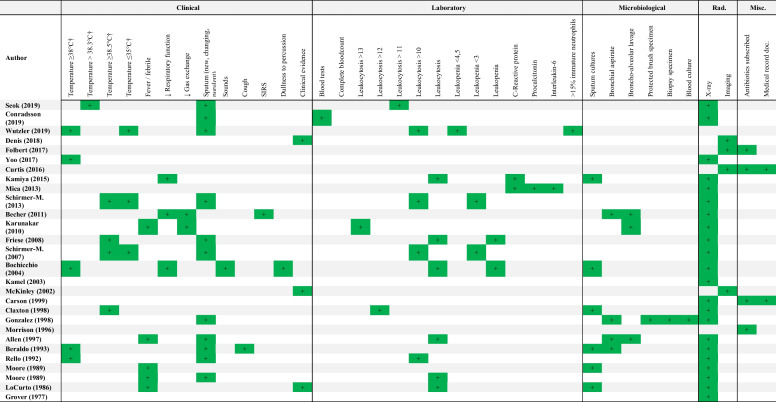
Non-guideline criteria used to diagnose hospital-acquired pneumonia in trauma patient research

†: ≤ and < , and ≥ and > are used interchangeably in this table when body temperatures are corresponding between studies

Abbreviations: *Misc* miscellaneous, *Rad* radiological, *SIRS* systemic inflammatory response syndrome, *↓* worsening

A detailed overview of the used criteria in all included studies was added in Supplemental Table 4.

### Methodological quality of included studies

The MINORS score for comparative studies ranged from 9 to 22 on a potential maximum score of 24. For non-comparative studies, the range was 4 to 9 out of 16. The minimum (9 vs. 4) and maximum scores (22 vs. 21) were not considerably different for studies with guideline and non-guideline criteria, respectively (Table 1; Supplemental Table 5).

## Discussion

This systematic review provides a general overview of criteria utilized in trauma patient research to diagnose hospital-acquired pneumonia. In only 46 out of 5749 original studies, well-defined criteria were reported, either pre-defined by published guidelines or clear non-guideline criteria. Forty-eight unique criteria were presented and clustered into five categories: clinical, laboratory, microbiological, radiological, and miscellaneous.

In the 28 studies without pre-defined guideline criteria to diagnose HAP, 37 unique criteria were reported. The heterogeneity in the applied criteria can mainly be attributed to the vast diversity in clinical, laboratory, and microbiological thresholds. For example, when considering leukocyte count as an indicator of HAP, up to five different thresholds were reported, describing both an elevated and decreased leukocyte count as indicative of HAP. One could imagine that a lower cut-off point of leukocytosis (e.g., 10 × 10^9^/L versus 13 × 10^9^/L) may lead to a higher estimate of HAP cases in a research population. Similar threshold differences were observed for body temperature, including “fever” or “febrile” as subjective criteria.

Some studies cited established guidelines as a basis for diagnosis, but the authors added new criteria or deleted pre-defined criteria, thus introducing (potential) aggregate bias. For instance, four studies added “medical record documentation” or “start of antibiotic treatment” as a criterion to diagnose pneumonia in addition to guideline criteria [12, 28, 30, 61]. Also, several studies added specific criteria (e.g., hypothermia, worsening gas exchange, leukopenia, and bronchoalveolar lavage) to the ATS/IDSA criteria [12, 22, 34, 61, 64]. Though all are clinically relevant criteria, adjusting pre-defined criteria complicates the comparison of studies that use the same guidelines and increases bias.

Eighteen studies applied existing guideline criteria to diagnose HAP. However, five different guidelines were encountered, leading to a further decrease in uniformity. We encountered a similar variation in body temperature and leukocyte count cut-offs (Table 2) [7–9, 65–67]. However, the number of variations was lower for the pre-defined guideline criteria: three versus five cut-offs for body temperature and leukocytosis. The 2015 ATS/IDSA guideline contained no distinct thresholds for hyperthermia and leukocytosis, resulting in differences between studies that used this guideline (Table 2) [67]. Despite the attempts to generate uniformity in diagnosing HAP by creating and using guideline criteria, the abovementioned differences make it difficult to compare the available literature completely. Only five studies diagnosed HAP based on the exact same criteria.

Guidelines are continuously updated based on new insights and available literature. The earliest CDC guideline dates from 1988, and the most recent from 2018. During these 30 years, the criteria for pneumonia diagnosis have changed substantially. For example, the 1988 CDC criteria for pneumonia diagnosis were clinical, radiologic, or microbiologic, while body temperature was not included as a criterion [68]. However, the 2018 guideline provides a more elaborate set of clinical, radiological, microbiological, and laboratory criteria [7]. As a result, it is more difficult to compare older data sets to more recent studies. Full implementation of or compliance with guideline criteria in clinical practice is hardly feasible for two reasons: patient care and study design. Study subjects are patients; therefore, clinical examination and experience remain decisive in starting pneumonia treatment. The authors understand that an inconclusive or negative X-ray should not delay antibiotic treatment, and awaiting a microbial culture is not mandatory or necessary in seriously ill patients. Using guideline criteria for pneumonia diagnosis in retrospective studies might be impracticable. Also, uniform diagnostic criteria for pneumonia are hard to accomplish in database or registry studies. Nonetheless, these limitations should be mentioned when encountered.

Previously, review studies have been issued on lacking definitions in trauma research, such as fracture-related infections and non-unions of long bones [69–71]. To resolve a lack of definition, these review studies provide a basis for a consensus definition. Subsequently, Delphi method studies can be helpful in reaching consensus. Our study displays the wide variety of clinical criteria for HAP diagnosis in trauma research and exhibits how studies ought to be compared with caution. The comparison of results is essential in trauma patient research and for guidelines. Guideline issuers pursue workable and representable guidelines to help clinicians in decision-making. Continuous improvement is established with the results of clinical studies, for which comparability of results is necessary. Our results emphasize this importance. Though our study addressed a scientific problem rather than a clinical one, it can still impact day-to-day practice.

One established definition of HAP would improve the comparability of trauma research; expert consensus could be a solid foundation to start with. Given the complexity of trauma patients, any diagnostic definition should address potential issues and pitfalls to avoid overdiagnosis. Currently, hyperthermia is incorporated in all guidelines, and sputum and dyspnea (with or without worsening gas exchange) in all but one. Leukocytosis, a common marker for infection, is included in all guidelines. Microbiologic information and evidence of infection aid in diagnosis and treatment; the CDC and ECDC describe several types of respiratory cultures. Radiologic evidence of pneumonia—either radiographic or CT imaging—also supports a diagnosis. Expert consensus should incorporate these criteria. Nonetheless, hypo- or hyperthermia, dyspnea, and leukocytosis are also signs of the systemic inflammatory response syndrome, commonly observed in trauma patients and potentially complicating the diagnostic process [72]. Also, posttraumatic fever may have a non-infectious origin, such as neurogenic fever, and trauma is associated with an increased immune response, adding to the need for a dedicated leukocytosis threshold [73, 74]. Lastly, sputum can result from (severe) pulmonary contusion, though unlikely to be purulent [75]. We propose that a decision-making algorithm includes hyperthermia (≥ 38.5 ℃) and leukocytosis (> 12 × 10^9^/L) as major criteria, in addition to microbiologic and radiologic evidence. Dyspnea and (purulent) sputum should be considered minor criteria. Our considerations and recommendations serve as a basis for expert consensus.

Some limitations of this study should be considered. Firstly, PubMed/MEDLINE was the only search engine used in this study, which could result in an incomplete overview of applied clinical criteria for HAP diagnosis. However, the wide variety of clinical criteria and the difficult comparison between studies are evident in the current number of included studies. Secondly, our overview of reported diagnostic criteria did not consider the recommended combinations of these criteria. Not doing so resulted in a more comprehensible overview of used criteria and did not diminish the conclusion of this study.

## Conclusion

As few studies in trauma patient research report a clear, clinical definition of hospital-acquired pneumonia, results cannot be adequately compared. Moreover, the wide variety of non-guideline criteria and diversity in pre-defined guideline criteria do not facilitate proper comparison. Studies should at least report how a diagnosis was made, but preferably, they would use pre-defined guideline criteria for pneumonia diagnosis in a research setting. Ideally, one internationally accepted set of criteria is used to diagnose hospital-acquired pneumonia.

## Supplementary Information

Below is the link to the electronic supplementary material.

Supplementary file1 (DOCX 54 KB)

## Data Availability

No datasets were generated or analysed during the current study.

## Abbreviations

ATS: American Thoracic Society
BSAC: British Society for Antimicrobial Chemotherapy
CDC: Centers for Disease Control and Prevention
ECDC: European Center for Disease Prevention and Control
HAP: Hospital-acquired pneumonia
IDSA: Infectious Disease Society of America
SIR: Swedish Intensive Care Registry
VAP: Ventilator-associated pneumonia

## References

[1] Glance LG, Stone PW, Mukamel DB, Dick AW. Increases in mortality, length of stay, and cost associated with hospital-acquired infections in trauma patients. Arch Surg. 2011;146(7):794–801. 10.1001/archsurg.2011.41. 21422331 10.1001/archsurg.2011.41 PMC3336161

[2] Esnault P, Nguyen C, Bordes J, D’Aranda E, Montcriol A, Contargyris C, et al. Early-onset ventilator-associated pneumonia in patients with severe traumatic brain injury: incidence, risk factors, and consequences in cerebral oxygenation and outcome. Neurocrit Care. 2017;27(2):187–98. 10.1007/s12028-017-0397-4. 28432539 10.1007/s12028-017-0397-4

[3] Major JS, Welbourne J. Nosocomial infection in trauma intensive care. J Intensive Care Soc. 2015;16(3):193–8. 10.1177/1751143715579076. 28979409 10.1177/1751143715579076 PMC5606446

[4] Andermahr J, Greb A, Hensler T, Helling HJ, Bouillon B, Sauerland S, et al. Pneumonia in multiple injured patients: a prospective controlled trial on early prediction using clinical and immunological parameters. Inflamm Res. 2002;51(5):265–72. 12056515 10.1007/pl00000303

[5] Byun JH, Kim HY. Factors affecting pneumonia occurring to patients with multiple rib fractures. Korean J Thorac Cardiovasc Surg. 2013;46(2):130–4. 10.5090/kjtcs.2013.46.2.130. 23614099 10.5090/kjtcs.2013.46.2.130 PMC3631787

[6] Anand N, Kollef MH. The alphabet soup of pneumonia: CAP, HAP, HCAP, NHAP, and VAP. Semin Respir Crit Care Med. 2009;30(1):3–9. 10.1055/s-0028-1119803. 19199181 10.1055/s-0028-1119803

[7] Network NHS. Pneumonia (ventilator-associated [VAP] and nonventilator-associated pneumonia [PNEU]) event: Centre for Disease Control (CDC); 2021 [Available from: https://www.cdc.gov/nhsn/pdfs/pscmanual/6pscvapcurrent.pdf. Accessed 15 Dec 2020.

[8] Kalil AC, Metersky ML, Klompas M, Muscedere J, Sweeney DA, Palmer LB, et al. Management of adults with hospital-acquired and ventilator-associated pneumonia: 2016 clinical practice guidelines by the Infectious Diseases Society of America and the American Thoracic Society. Clin Infect Dis. 2016;63(5):e61–111. 10.1093/cid/ciw353. 27418577 10.1093/cid/ciw353 PMC4981759

[9] European Center for Disease Prevention and Control (ECDC). Point prevalence survey of healthcare-associated infections and antimicrobial use in European acute care hospitals: full-scale survey and codebook. Stockholm: ECDC; 2012.

[10] Rotstein C, Evans G, Born A, Grossman R, Light RB, Magder S, et al. Clinical practice guidelines for hospital-acquired pneumonia and ventilator-associated pneumonia in adults. Can J Infect Dis Med Microbiol. 2008;19(1):19–53. 10.1155/2008/593289. 19145262 10.1155/2008/593289 PMC2610276

[11] Koenig SM, Truwit JD. Ventilator-associated pneumonia: diagnosis, treatment, and prevention. Clin Microbiol Rev. 2006;19(4):637–57. 10.1128/CMR.00051-05. 17041138 10.1128/CMR.00051-05 PMC1592694

[12] Yang Y, Young JB, Schermer CR, Utter GH. Use of ketorolac is associated with decreased pneumonia following rib fractures. Am J Surg. 2014;207(4):566–72. 10.1016/j.amjsurg.2013.05.011. 24112670 10.1016/j.amjsurg.2013.05.011 PMC4393822

[13] Andermahr J, Hensler T, Sauerland S, Greb A, Helling HJ, Prokop A, et al. Risk factors for the development of pneumonia in multiple injured patients. Results of a prospective clinical trial. Unfallchirurg. 2003;106(5):392–7. 10.1007/s00113-003-0592-y. 12750813 10.1007/s00113-003-0592-y PMC7096010

[14] McInnes MDF, Moher D, Thombs BD, McGrath TA, Bossuyt PM, P-DTAG, et al. Preferred Reporting Items for a Systematic Review and Meta-analysis of Diagnostic Test Accuracy Studies: the PRISMA-DTA statement. JAMA. 2018;319(4):388–96. 10.1001/jama.2017.19163. 29362800 10.1001/jama.2017.19163

[15] Ouzzani M, Hammady H, Fedorowicz Z, Elmagarmid A. Rayyan-a web and mobile app for systematic reviews. Syst Rev. 2016;5(1):210. 10.1186/s13643-016-0384-4. 27919275 10.1186/s13643-016-0384-4 PMC5139140

[16] Slim K, Nini E, Forestier D, Kwiatkowski F, Panis Y, Chipponi J. Methodological index for non-randomized studies (minors): development and validation of a new instrument. ANZ J Surg. 2003;73(9):712–6. 10.1046/j.1445-2197.2003.02748.x. 12956787 10.1046/j.1445-2197.2003.02748.x

[17] Wutzler S, Blasius FM, Stormann P, Lustenberger T, Frink M, Maegele M, et al. Pneumonia in severely injured patients with thoracic trauma: results of a retrospective observational multi-centre study. Scand J Trauma Resusc Emerg Med. 2019;27(1):31. 10.1186/s13049-019-0608-4. 30871601 10.1186/s13049-019-0608-4 PMC6419484

[18] Warren C, Medei MK, Wood B, Schutte D. A nurse-driven oral care protocol to reduce hospital-acquired pneumonia. Am J Nurs. 2019;119(2):44–51. 10.1097/01.NAJ.0000553204.21342.01. 30681478 10.1097/01.NAJ.0000553204.21342.01

[19] Seok J, Cho HM, Kim HH, Kim JH, Huh U, Kim HB, et al. Chest trauma scoring systems for predicting respiratory complications in isolated rib fracture. J Surg Res. 2019;244:84–90. 10.1016/j.jss.2019.06.009. 31279998 10.1016/j.jss.2019.06.009

[20] Conradsson D, Phillips J, Nizeyimana E, Hilliar C, Joseph C. Risk indicators of length of acute hospital stay after traumatic spinal cord injury in South Africa: a prospective, population-based study. Spinal Cord. 2019;57(9):763–9. 10.1038/s41393-019-0286-0. 31053775 10.1038/s41393-019-0286-0

[21] Yadollahi M, Kashkooe A, Feyzi M, Bornapour S. Risk factors of mortality in nosocomial infected traumatic patients in a trauma referral center in south of Iran. Chin J Traumatol. 2018;21(5):267–72. 10.1016/j.cjtee.2018.03.002. 29929766 10.1016/j.cjtee.2018.03.002 PMC6235789

[22] Guo C, Lei M, Wang Y, Hua L, Xue S, Yu D, et al. Oral administration of probiotic Lactobacillus casei Shirota decreases pneumonia and increases pulmonary functions after single rib fracture: a randomized double-blind, placebo-controlled clinical trial. J Food Sci. 2018;83(8):2222–6. 10.1111/1750-3841.14220. 30020533 10.1111/1750-3841.14220

[23] Djuric O, Markovic-Denic L, Jovanovic B, Bumbasirevic V. Agreement between CDC/NHSN surveillance definitions and ECDC criteria in diagnosis of healthcare-associated infections in Serbian trauma patients. PLoS One. 2018;13(10):e0204893. 10.1371/journal.pone.0204893. 30286119 10.1371/journal.pone.0204893 PMC6171870

[24] Denis AR, Feldman D, Thompson C, Mac-Thiong JM. Prediction of functional recovery six months following traumatic spinal cord injury during acute care hospitalization. J Spinal Cord Med. 2018;41(3):309–17. 10.1080/10790268.2017.1279818. 28198660 10.1080/10790268.2017.1279818 PMC6055948

[25] Yoo JH, Kim KT, Kim TY, Hwang JH, Chang JD. Postoperative fever after hemiarthroplasty in elderly patients over 70 years of age with displaced femoral neck fracture: necessity of routine workup? Injury. 2017;48(2):441–6. 10.1016/j.injury.2016.12.013. 28040259 10.1016/j.injury.2016.12.013

[26] Folbert EC, Hegeman JH, Gierveld R, van Netten JJ, Velde DV, Ten Duis HJ, et al. Complications during hospitalization and risk factors in elderly patients with hip fracture following integrated orthogeriatric treatment. Arch Orthop Trauma Surg. 2017;137(4):507–15. 10.1007/s00402-017-2646-6. 28233062 10.1007/s00402-017-2646-6

[27] Curtis K, Asha SE, Unsworth A, Lam M, Goldsmith H, Langcake M, et al. ChIP: an early activation protocol for isolated blunt chest injury improves outcomes, a retrospective cohort study. Australas Emerg Nurs J. 2016;19(3):127–32. 10.1016/j.aenj.2016.06.002. 27448460 10.1016/j.aenj.2016.06.002

[28] Yun HC, Weintrob AC, Conger NG, Li P, Lu D, Tribble DR, et al. Healthcare-associated pneumonia among U.S. combat casualties, 2009 to 2010. Mil Med. 2015;180(1):104–10. 10.7205/MILMED-D-14-00209. 25562865 10.7205/MILMED-D-14-00209 PMC4286028

[29] Kamiya K, Koda M, Furuya T, Kato K, Takahashi H, Sakuma T, et al. Neuroprotective therapy with granulocyte colony-stimulating factor in acute spinal cord injury: a comparison with high-dose methylprednisolone as a historical control. Eur Spine J. 2015;24(5):963–7. 10.1007/s00586-014-3373-0. 24961222 10.1007/s00586-014-3373-0

[30] Landeen C, Smith HL. Examination of pneumonia risks and risk levels in trauma patients with pulmonary contusion. J Trauma Nurs. 2014;21(2):41–9. 10.1097/JTN.0000000000000029. 24614291 10.1097/JTN.0000000000000029

[31] Schirmer-Mikalsen K, Moen KG, Skandsen T, Vik A, Klepstad P. Intensive care and traumatic brain injury after the introduction of a treatment protocol: a prospective study. Acta Anaesthesiol Scand. 2013;57(1):46–55. 10.1111/j.1399-6576.2012.02785.x. 23095138 10.1111/j.1399-6576.2012.02785.x

[32] Mica L, Keller C, Vomela J, Trentz O, Plecko M, Keel MJ. Obesity and overweight as a risk factor for pneumonia in polytrauma patients: a retrospective cohort study. J Trauma Acute Care Surg. 2013;75(4):693–8. 10.1097/TA.0b013e31829a0bdd. 24064885 10.1097/TA.0b013e31829a0bdd

[33] Hyllienmark P, Brattstrom O, Larsson E, Martling CR, Petersson J, Oldner A. High incidence of post-injury pneumonia in intensive care-treated trauma patients. Acta Anaesthesiol Scand. 2013;57(7):848–54. 10.1111/aas.12111. 23550742 10.1111/aas.12111

[34] Yeung L, Miraflor E, Strumwasser A, Sadeghi P, Victorino GP. Does gastric volume in trauma patients identify a population at risk for developing pneumonia and poor outcomes? J Surg Res. 2012;178(2):874–8. 10.1016/j.jss.2012.07.067. 22917669 10.1016/j.jss.2012.07.067

[35] Hakim SM, Latif FS, Anis SG. Comparison between lumbar and thoracic epidural morphine for severe isolated blunt chest wall trauma: a randomized open-label trial. J Anesth. 2012;26(6):836–44. 10.1007/s00540-012-1424-4. 22674157 10.1007/s00540-012-1424-4

[36] Strumwasser A, Chu E, Yeung L, Miraflor E, Sadjadi J, Victorino GP. A novel CT volume index score correlates with outcomes in polytrauma patients with pulmonary contusion. J Surg Res. 2011;170(2):280–5. 10.1016/j.jss.2011.03.022. 21601877 10.1016/j.jss.2011.03.022

[37] Becher RD, Hoth JJ, Neff LP, Rebo JJ, Martin RS, Miller PR. Multidrug-resistant pathogens and pneumonia: comparing the trauma and surgical intensive care units. Surg Infect (Larchmt). 2011;12(4):267–72. 10.1089/sur.2010.052. 21524206 10.1089/sur.2010.052

[38] Karunakar MA, Staples KS. Does stress-induced hyperglycemia increase the risk of perioperative infectious complications in orthopaedic trauma patients? J Orthop Trauma. 2010;24(12):752–6. 10.1097/BOT.0b013e3181d7aba5. 21076247 10.1097/BOT.0b013e3181d7aba5

[39] Garcia-Alvarez F, Al-Ghanem R, Garcia-Alvarez I, Lopez-Baisson A, Bernal M. Risk factors for postoperative infections in patients with hip fracture treated by means of Thompson arthroplasty. Arch Gerontol Geriatr. 2010;50(1):51–5. 10.1016/j.archger.2009.01.009. 19233490 10.1016/j.archger.2009.01.009

[40] Friese RS, Sperry JL, Phelan HA, Gentilello LM. The use of leukoreduced red blood cell products is associated with fewer infectious complications in trauma patients. Am J Surg. 2008;196(1):56–61. 10.1016/j.amjsurg.2007.08.063. 18513694 10.1016/j.amjsurg.2007.08.063

[41] Schlosser HG, Volk HD, Splettstosser G, Brock M, Woiciechowsky C. A new qualitative interleukin-6 bedside test can predict pneumonia in patients with severe head injury–comparison to the standard Immulite test and a semiquantitative bedside test. J Neurosurg Anesthesiol. 2007;19(1):5–9. 10.1097/01.ana.0000211026.18926.89. 17198094 10.1097/01.ana.0000211026.18926.89

[42] Schirmer-Mikalsen K, Vik A, Gisvold SE, Skandsen T, Hynne H, Klepstad P. Severe head injury: control of physiological variables, organ failure and complications in the intensive care unit. Acta Anaesthesiol Scand. 2007;51(9):1194–201. 10.1111/j.1399-6576.2007.01372.x. 17711565 10.1111/j.1399-6576.2007.01372.x

[43] Kamel HK, Iqbal MA, Mogallapu R, Maas D, Hoffmann RG. Time to ambulation after hip fracture surgery: relation to hospitalization outcomes. J Gerontol A Biol Sci Med Sci. 2003;58(11):1042–5. 10.1093/gerona/58.11.m1042. 14630887 10.1093/gerona/58.11.m1042

[44] McKinley WO, Tewksbury MA, Godbout CJ. Comparison of medical complications following nontraumatic and traumatic spinal cord injury. J Spinal Cord Med. 2002;25(2):88–93. 10.1080/10790268.2002.11753607. 12137222 10.1080/10790268.2002.11753607

[45] Carson JL, Altman DG, Duff A, Noveck H, Weinstein MP, Sonnenberg FA, et al. Risk of bacterial infection associated with allogeneic blood transfusion among patients undergoing hip fracture repair. Transfusion. 1999;39(7):694–700. 10.1046/j.1537-2995.1999.39070694.x. 10413276 10.1046/j.1537-2995.1999.39070694.x

[46] Gonzalez RP, Holevar MR. Role of prophylactic antibiotics for tube thoracostomy in chest trauma. Am Surg. 1998;64(7):617–20 (discussion 20-1). 9655270

[47] Claxton AR, Wong DT, Chung F, Fehlings MG. Predictors of hospital mortality and mechanical ventilation in patients with cervical spinal cord injury. Can J Anaesth. 1998;45(2):144–9. 10.1007/BF03013253. 9512849 10.1007/BF03013253

[48] Morrison JE, Wisner DH, Bodai BI. Complications after negative laparotomy for trauma: long-term follow-up in a health maintenance organization. J Trauma. 1996;41(3):509–13. 10.1097/00005373-199609000-00021. 8810972 10.1097/00005373-199609000-00021

[49] Renz BM, Feliciano DV. Unnecessary laparotomies for trauma: a prospective study of morbidity. J Trauma. 1995;38(3):350–6. 10.1097/00005373-199503000-00007. 7897713 10.1097/00005373-199503000-00007

[50] Nichols RL, Smith JW, Muzik AC, Love EJ, McSwain NE, Timberlake G, et al. Preventive antibiotic usage in traumatic thoracic injuries requiring closed tube thoracostomy. Chest. 1994;106(5):1493–8. 10.1378/chest.106.5.1493. 7956409 10.1378/chest.106.5.1493

[51] Beraldo PS, Neves EG, Alves CM, Khan P, Cirilo AC, Alencar MR. Pyrexia in hospitalised spinal cord injury patients. Paraplegia. 1993;31(3):186–91. 10.1038/sc.1993.35. 8479785 10.1038/sc.1993.35

[52] Moore FA, Moore EE, Jones TN, McCroskey BL, Peterson VM. TEN versus TPN following major abdominal trauma–reduced septic morbidity. J Trauma. 1989;29(7):916–22. 10.1097/00005373-198907000-00003. (discussion 22-3). 2501509 10.1097/00005373-198907000-00003

[53] Moore FA, Moore EE, Ammons LA, McCroskey BL. Presumptive antibiotics for penetrating abdominal wounds. Surg Gynecol Obstet. 1989;169(2):99–103. 2667180

[54] LoCurto JJ Jr, Tischler CD, Swan KG, Rocko JM, Blackwood JM, Griffin CC, et al. Tube thoracostomy and trauma–antibiotics or not? J Trauma. 1986;26(12):1067–72. 10.1097/00005373-198612000-00001. 3795301 10.1097/00005373-198612000-00001

[55] Grover FL, Richardson JD, Fewel JG, Arom KV, Webb GE, Trinkle JK. Prophylactic antibiotics in the treatment of penetrating chest wounds. A prospective double-blind study. J Thorac Cardiovasc Surg. 1977;74(4):528–36. 333188

[56] Allen GS, Cox CS Jr, Moore FA, Duke JH, Andrassy RJ. Pulmonary contusion: are children different? J Am Coll Surg. 1997;185(3):229–33. 9291398

[57] Becher RD, Hoth JJ, Rebo JJ, Kendall JL, Miller PR. Locally derived versus guideline-based approach to treatment of hospital-acquired pneumonia in the trauma intensive care unit. Surg Infect (Larchmt). 2012;13(6):352–9. 10.1089/sur.2011.056. 23268613 10.1089/sur.2011.056

[58] Beghi G, De Tanti A, Serafini P, Bertolino C, Celentano A, Taormina G. Monitoring of hospital acquired pneumonia in patients with severe brain injury on first access to intensive neurological rehabilitation: first year of observation. Monaldi Arch Chest Dis. 2018;88(1):888. 10.4081/monaldi.2018.888. 29741076 10.4081/monaldi.2018.888

[59] Bochicchio GV, Joshi M, Bochicchio K, Tracy K, Scalea TM. A time-dependent analysis of intensive care unit pneumonia in trauma patients. J Trauma. 2004;56(2):296–301. 10.1097/01.TA.0000109857.22312.DF. (discussion -3). 14960971 10.1097/01.TA.0000109857.22312.DF

[60] Bozorgzadeh A, Pizzi WF, Barie PS, Khaneja SC, LaMaute HR, Mandava N, et al. The duration of antibiotic administration in penetrating abdominal trauma. Am J Surg. 1999;177(2):125–31. 10.1016/s0002-9610(98)00317-1. 10204554 10.1016/s0002-9610(98)00317-1

[61] Ewan VC, Sails AD, Walls AW, Rushton S, Newton JL. Dental and microbiological risk factors for hospital-acquired pneumonia in non-ventilated older patients. PLoS One. 2015;10(4):e0123622. 10.1371/journal.pone.0123622. 25923662 10.1371/journal.pone.0123622 PMC4414413

[62] Giamberardino HI, Cesario EP, Carmes ER, Mulinari RA. Risk factors for nosocomial infection in trauma patients. Braz J Infect Dis. 2007;11(2):285–9. 10.1590/s1413-86702007000200024. 17625779 10.1590/s1413-86702007000200024

[63] Rello J, Ausina V, Castella J, Net A, Prats G. Nosocomial respiratory tract infections in multiple trauma patients. Influence of level of consciousness with implications for therapy. Chest. 1992;102(2):525–9. 10.1378/chest.102.2.525. 1643942 10.1378/chest.102.2.525

[64] Worrall CL, Anger BP, Simpson KN, Leon SM. Impact of a hospital-acquired/ventilator-associated/healthcare-associated pneumonia practice guideline on outcomes in surgical trauma patients. J Trauma. 2010;68(2):382–6. 10.1097/TA.0b013e318197bc74. 19935109 10.1097/TA.0b013e318197bc74

[65] (SIR) SICR. [SIR's guideline for registration of adverse events and complications in intensive care in Sweden] 2019 [21.0:[Available from: https://www.icuregswe.org/globalassets/riktlinjer/komplikationer_21.pdf. Accessed 15 Dec 2020.

[66] Masterton RG, Galloway A, French G, Street M, Armstrong J, Brown E, et al. Guidelines for the management of hospital-acquired pneumonia in the UK: report of the working party on hospital-acquired pneumonia of the British Society for Antimicrobial Chemotherapy. J Antimicrob Chemother. 2008;62(1):5–34. 10.1093/jac/dkn162. 18445577 10.1093/jac/dkn162 PMC7110234

[67] Society AT, America IDSo. Guidelines for the management of adults with hospital-acquired, ventilator-associated, and healthcare-associated pneumonia. Am J Respir Crit Care Med. 2005;171(4):388–416. 10.1164/rccm.200405-644ST. 15699079 10.1164/rccm.200405-644ST

[68] Garner JS, Jarvis WR, Emori TG, Horan TC, Hughes JM. CDC definitions for nosocomial infections, 1988. Am J Infect Control. 1988;16(3):128–40. 10.1016/0196-6553(88)90053-3. 2841893 10.1016/0196-6553(88)90053-3

[69] Metsemakers WJ, Kuehl R, Moriarty TF, Richards RG, Verhofstad MHJ, Borens O, et al. Infection after fracture fixation: current surgical and microbiological concepts. Injury. 2018;49(3):511–22. 10.1016/j.injury.2016.09.019. 27639601 10.1016/j.injury.2016.09.019

[70] Wittauer M, Burch MA, McNally M, Vandendriessche T, Clauss M, Della Rocca GJ, et al. Definition of long-bone nonunion: a scoping review of prospective clinical trials to evaluate current practice. Injury. 2021;52(11):3200–5. 10.1016/j.injury.2021.09.008. 34531088 10.1016/j.injury.2021.09.008

[71] Metsemakers WJ, Morgenstern M, McNally MA, Moriarty TF, McFadyen I, Scarborough M, et al. Fracture-related infection: a consensus on definition from an international expert group. Injury. 2018;49(3):505–10. 10.1016/j.injury.2017.08.040. 28867644 10.1016/j.injury.2017.08.040

[72] Chakraborty RK, Burns B. Systemic inflammatory response syndrome. Treasure Island (FL): StatPearls Publisher; 2022. 31613449

[73] Thompson HJ, Pinto-Martin J, Bullock MR. Neurogenic fever after traumatic brain injury: an epidemiological study. J Neurol Neurosurg Psychiatry. 2003;74(5):614–9. 10.1136/jnnp.74.5.614. 12700304 10.1136/jnnp.74.5.614 PMC1738450

[74] Lord JM, Midwinter MJ, Chen YF, Belli A, Brohi K, Kovacs EJ, et al. The systemic immune response to trauma: an overview of pathophysiology and treatment. Lancet. 2014;384(9952):1455–65. 10.1016/S0140-6736(14)60687-5. 25390327 10.1016/S0140-6736(14)60687-5 PMC4729362

[75] Ganie FA, Lone H, Lone GN, Wani ML, Singh S, Dar AM, et al. Lung contusion: a clinico-pathological entity with unpredictable clinical course. Bull Emerg Trauma. 2013;1(1):7–16. 27162815 PMC4771236

