# Supplementary material for: Definitions of hospital-acquired pneumonia in trauma research: a systematic review
Source: Eur J Trauma Emerg Surg. 2024 Mar 28;50(5):2005–15. doi: 10.1007/s00068-024-02509-8 (PMC11599634; doi:10.1007/s00068-024-02509-8)
Supplement: Supplementary file 1 — Supplementary file1 (DOCX 54 KB) [file 68_2024_2509_MOESM1_ESM.docx]

| **Supplemental Table 1.** PubMed search syntax. |
| --- |
| “Pneumonia”[MeSH] OR Pneumonia[Title/Abstract] OR Pneumonias[Title/Abstract] OR “Lung Inflammation*”[Title/Abstract] OR Pneumonitis[Title/Abstract] OR Pneumonitides[Title/Abstract] OR “Pulmonary Inflammation*”[Title/Abstract] OR “Lower Respiratory Tract Infection*”[Title/Abstract]  AND  “Clinical criteria”[Title/Abstract] OR “Clinical Criterium”[Title/Abstract] OR Definition*[Title/Abstract] OR Define[Title/Abstract] OR “Clinical Protocol*”[Title/Abstract] OR “Treatment Protocol*”[Title/Abstract] OR “Clinical Research Protocol*”[Title/Abstract] OR Incidence[MeSH] OR Incidence[Title/Abstract] OR Prevalence[MeSH] OR Prevalence*[Title/Abstract] OR Epidemiology[Title/Abstract] OR “Risk Factors”[MeSH] OR Risk Factor*[Title/Abstract] OR Identification[Title/Abstract] OR “Prevention and Control”[Subheading] OR Prevention[Title/Abstract] OR Preventing[Title/Abstract] OR Prophylaxis[Title/Abstract] OR Prediction[Title/Abstract] OR Predicting[Title/Abstract] OR Predictor*[Title/Abstract]  AND  "Injuries"[Subheading] OR Trauma[Title/Abstract] OR Traumatic[Title/Abstract] OR Posttrauma[Title/Abstract] OR Post-trauma[Title/Abstract] OR Posttraumatic[Title/Abstract] OR Post-traumatic[Title/Abstract] OR Injury[Title/Abstract] OR Injuries[Title/Abstract] OR Injured[Title/Abstract] OR Postinjury[Title/Abstract] OR Post-injury[Title/Abstract] OR Polytrauma[Title/Abstract] OR Poly-trauma[Title/Abstract] OR “Wounds and Injuries”[MeSH] OR Wound[Title/Abstract] OR Wounds[Title/Abstract] OR Rupture[Title/Abstract] OR Ruptures[Title/Abstract] OR Fracture*[Title/Abstract] OR Head-Trauma*[Title/Abstract]  NOT  animals[MeSH] NOT humans[MeSH] |

| **Supplemental Table 2.** Bibliography of articles not included in qualitative synthesis because of the absence of a definition or stating a non-clinical definition of hospital-acquired pneumonia. |
| --- |
| *Articles that stated specific ICD-codes*  Glance, L. G., Stone, P. W., Mukamel, D. B., & Dick, A. W. (2011). Increases in mortality, length of stay, and cost associated with hospital-acquired infections in trauma patients. *Arch Surg, 146*(7), 794-801. doi:10.1001/archsurg.2011.41  Hadjibashi, A. A., Berry, C., Ley, E. J., Bukur, M., Mirocha, J., Stolpner, D., & Salim, A. (2012). Alcohol is associated with a lower pneumonia rate after traumatic brain injury. *J Surg Res, 173*(2), 212-215. doi:10.1016/j.jss.2011.05.029  Ho, S. W., Teng, Y. H., Yang, S. F., Yeh, H. W., Wang, Y. H., Chou, M. C., & Yeh, C. B. (2017). Risk of pneumonia in patients with isolated minor rib fractures: a nationwide cohort study. *BMJ Open, 7*(1), e013029. doi:10.1136/bmjopen-2016-013029  Ingraham, A. M., Xiong, W., Hemmila, M. R., Shafi, S., Goble, S., Neal, M. L., & Nathens, A. B. (2010). The attributable mortality and length of stay of trauma-related complications: a matched cohort study. *Ann Surg, 252*(2), 358-362. doi:10.1097/SLA.0b013e3181e623bf  Janus, T. J., Vaughan-Sarrazin, M. S., Baker, L. J., & Smith, H. L. (2012). Predictors of pneumonia in trauma patients with pulmonary contusion. *J Trauma Nurs, 19*(3), 139-147. doi:10.1097/JTN.0b013e318261d1e2  Myers, A. H., Robinson, E. G., Van Natta, M. L., Michelson, J. D., Collins, K., & Baker, S. P. (1991). Hip fractures among the elderly: factors associated with in-hospital mortality. *Am J Epidemiol, 134*(10), 1128-1137. doi:10.1093/oxfordjournals.aje.a116016  Neuhaus, V., Swellengrebel, C. H., Bossen, J. K., & Ring, D. (2013). What are the factors influencing outcome among patients admitted to a hospital with a proximal humeral fracture? *Clin Orthop Relat Res, 471*(5), 1698-1706. doi:10.1007/s11999-013-2876-z  Prin, M., & Li, G. (2016). Complications and in-hospital mortality in trauma patients treated in intensive care units in the United States, 2013. *Inj Epidemiol, 3*(1), 18. doi:10.1186/s40621-016-0084-5  Raab, A. M., Krebs, J., Perret, C., Michel, F., Hopman, M. T., & Mueller, G. (2016). Maximum Inspiratory Pressure is a Discriminator of Pneumonia in Individuals With Spinal-Cord Injury. *Respir Care, 61*(12), 1636-1643. doi:10.4187/respcare.04818 |
| *Articles that did not state a definition or specific ICD-codes*  Aljerian, N., Alhaidar, S., Alothman, A., AlJohi, W., Albaqami, F. A., & Alghnam, S. A. (2018). Association between the mode of transport and in-hospital medical complications in trauma patients: findings from a level-I trauma center in Saudi Arabia. *Ann Saudi Med, 38*(1), 8-14. doi:10.5144/0256-4947.2018.8  Bender, J. S., Geller, E. R., & Wilson, R. F. (1989). Intra-abdominal sepsis following liver trauma. *J Trauma, 29*(8), 1140-1144; discussion 1144-1145. doi:10.1097/00005373-198908000-00012  Bongard, F. S., & Lim, R. C., Jr. (1985). Surgery of the traumatized spleen. *World J Surg, 9*(3), 391-397. doi:10.1007/BF01655273  Brasel, K. J., Guse, C. E., Layde, P., & Weigelt, J. A. (2006). Rib fractures: relationship with pneumonia and mortality. *Crit Care Med, 34*(6), 1642-1646. doi:10.1097/01.CCM.0000217926.40975.4B  Brown, C. A., Boling, J., Manson, M., Owens, T., & Zura, R. (2012). Relation between prefracture characteristics and perioperative complications in the elderly adult patient with hip fracture. *South Med J, 105*(6), 306-310. doi:10.1097/SMJ.0b013e3182574bfd  Browne, J. A., Cook, C., Olson, S. A., & Bolognesi, M. P. (2009). Resident duty-hour reform associated with increased morbidity following hip fracture. *J Bone Joint Surg Am, 91*(9), 2079-2085. doi:10.2106/JBJS.H.01240  Browne, J. A., Pietrobon, R., & Olson, S. A. (2009). Hip fracture outcomes: does surgeon or hospital volume really matter? *J Trauma, 66*(3), 809-814. doi:10.1097/TA.0b013e31816166bb  Brundage, S. I., McGhan, R., Jurkovich, G. J., Mack, C. D., & Maier, R. V. (2002). Timing of femur fracture fixation: effect on outcome in patients with thoracic and head injuries. *J Trauma, 52*(2), 299-307. doi:10.1097/00005373-200202000-00016  Carver, T. W., Milia, D. J., Somberg, C., Brasel, K., & Paul, J. (2015). Vital capacity helps predict pulmonary complications after rib fractures. *J Trauma Acute Care Surg, 79*(3), 413-416. doi:10.1097/TA.0000000000000744  Chou, S. E., Rau, C. S., Tsai, Y. C., Hsu, S. Y., Hsieh, H. Y., & Hsieh, C. H. (2019). Risk factors and complications contributing to mortality in elderly patients with fall-induced femoral fracture: A cross-sectional analysis based on trauma registry data of 2,407 patients. *Int J Surg, 66*, 48-52. doi:10.1016/j.ijsu.2019.04.010  Cichos, K. H., Churchill, J. L., Phillips, S. G., Watson, S. L., McGwin, G., Jr., Ghanem, E. S., & Ponce, B. A. (2018). Metabolic syndrome and hip fracture: Epidemiology and perioperative outcomes. *Injury, 49*(11), 2036-2041. doi:10.1016/j.injury.2018.09.012  Clond, M. A., Mirocha, J., Singer, M. B., Bukur, M., Salim, A., Marguiles, D. R., & Ley, E. J. (2011). Gender influences outcomes in trauma patients with elevated systolic blood pressure. *Am J Surg, 202*(6), 823-827; discussion 828. doi:10.1016/j.amjsurg.2011.06.044  Colon-Emeric, C. S., Mesenbrink, P., Lyles, K. W., Pieper, C. F., Boonen, S., Delmas, P., . . . Magaziner, J. (2010). Potential mediators of the mortality reduction with zoledronic acid after hip fracture. *J Bone Miner Res, 25*(1), 91-97. doi:10.1359/jbmr.090704  Deakin, D. E., Boulton, C., & Moran, C. G. (2007). Mortality and causes of death among patients with isolated limb and pelvic fractures. *Injury, 38*(3), 312-317. doi:10.1016/j.injury.2006.09.024  Dente, C. J., Tyburski, J., Wilson, R. F., Collinge, J., Steffes, C., & Carlin, A. (2000). Ostomy as a risk factor for posttraumatic infection in penetrating colonic injuries: univariate and multivariate analyses. *J Trauma, 49*(4), 628-634; discussion 634-627. doi:10.1097/00005373-200010000-00009  Dhall, S. S., Yue, J. K., Winkler, E. A., Mummaneni, P. V., Manley, G. T., & Tarapore, P. E. (2017). Morbidity and Mortality Associated with Surgery of Traumatic C2 Fractures in Octogenarians. *Neurosurgery, 80*(6), 854-862. doi:10.1093/neuros/nyw168  Dick, A. G., Davenport, D., Bansal, M., Burch, T. S., & Edwards, M. R. (2017). Hip Fractures in Centenarians: Has Care Improved in the National Hip Fracture Database Era? *Geriatr Orthop Surg Rehabil, 8*(3), 161-165. doi:10.1177/2151458517722104  Ekstrom, W., Samuelsson, B., Ponzer, S., Cederholm, T., Thorngren, K. G., & Hedstrom, M. (2015). Sex effects on short-term complications after hip fracture: a prospective cohort study. *Clin Interv Aging, 10*, 1259-1266. doi:10.2147/CIA.S80100  Facchinello, Y., Beausejour, M., Richard-Denis, A., Thompson, C., & Mac-Thiong, J. M. (2017). The use of regression tree analysis for predicting the functional outcome following traumatic spinal cord injury. *J Neurotrauma*. doi:10.1089/neu.2017.5321  Freedland, M., Wilson, R. F., Bender, J. S., & Levison, M. A. (1990). The management of flail chest injury: factors affecting outcome. *J Trauma, 30*(12), 1460-1468. doi:10.1097/00005373-199012000-00004  Glennie, R. A., Ailon, T., Yang, K., Batke, J., Fisher, C. G., Dvorak, M. F., . . . Street, J. T. (2015). Incidence, impact, and risk factors of adverse events in thoracic and lumbar spine fractures: an ambispective cohort analysis of 390 patients. *Spine J, 15*(4), 629-637. doi:10.1016/j.spinee.2014.11.016  Gold, A., Sever, R., Lerman, Y., Salai, M., & Justo, D. (2012). Admission Norton scale scores (ANSS) and postoperative complications following hip fracture surgery in the elderly. *Arch Gerontol Geriatr, 55*(1), 173-176. doi:10.1016/j.archger.2011.07.004  Gonzalez, K. W., Ghneim, M. H., Kang, F., Jupiter, D. C., Davis, M. L., & Regner, J. L. (2015). A pilot single-institution predictive model to guide rib fracture management in elderly patients. *J Trauma Acute Care Surg, 78*(5), 970-975. doi:10.1097/TA.0000000000000619  Guth, A. A., Hofstetter, S. R., & Pachter, H. L. (1996). Human immunodeficiency virus and the trauma patient: factors influencing postoperative infectious complications. *J Trauma, 41*(2), 251-255; discussion 255-256. doi:10.1097/00005373-199608000-00009  Haider, A. H., Gupta, S., Zogg, C. K., Kisat, M. T., Schupper, A., Efron, D. T., . . . Cornwell, E. E., 3rd. (2015). Beyond incidence: Costs of complications in trauma and what it means for those who pay. *Surgery, 158*(1), 96-103. doi:10.1016/j.surg.2015.02.015  Hoffmann C, Spiegl UJ, Hauck S, Buhren V, Gonschorek O. [What is the effect of ventral thoracoscopic spondylodesis (VTS) on elderly patients and what is the medium-term outcome?]. Z Orthop Unfall. 2013;151(3):257-63.  Ito, Y., Sugimoto, Y., Tomioka, M., Kai, N., & Tanaka, M. (2009). Does high dose methylprednisolone sodium succinate really improve neurological status in patient with acute cervical cord injury?: a prospective study about neurological recovery and early complications. *Spine (Phila Pa 1976), 34*(20), 2121-2124. doi:10.1097/BRS.0b013e3181b613c7  Kalil, M., & Amaral, I. M. (2016). Epidemiological evaluation of hepatic trauma victims undergoing surgery. *Rev Col Bras Cir, 43*(1), 22-27. doi:10.1590/0100-69912016001006  Kaneko, T. M., Foley, R. N., Gilbertson, D. T., & Collins, A. J. (2007). Clinical epidemiology of long-bone fractures in patients receiving hemodialysis. *Clin Orthop Relat Res, 457*, 188-193. doi:10.1097/BLO.0b013e318031465b  le Roux, J. C., & Dunn, R. N. (2005). Gunshot injuries of the spine--a review of 49 cases managed at the Groote Schuur Acute Spinal Cord Injury Unit. *S Afr J Surg, 43*(4), 165-168. Retrieved from https://www.ncbi.nlm.nih.gov/pubmed/16440591  Lee, J. M., Herrera-Escobar, J., Apoj, M., Al Rafai, S. S., Han, K., Nehra, D., . . . Kaafarani, H. M. A. (2019). The impact of in-hospital complications on the long-term functional outcome of trauma patients: A multicenter study. *Surgery, 166*(3), 398-402. doi:10.1016/j.surg.2019.04.026  Loewenstern, J., Kessler, R. A., & Caridi, J. (2019). Diabetes Comorbidity Increases Risk of Postoperative Complications in Traumatic Thoracic Vertebral Fracture Repair: A Propensity Score Matched Analysis. *World Neurosurg, 121*, e792-e797. doi:10.1016/j.wneu.2018.09.225  Marion, T. E., Rivers, C. S., Kurban, D., Cheng, C. L., Fallah, N., Batke, J., . . . Street, J. T. (2017). Previously Identified Common Post-Injury Adverse Events in Traumatic Spinal Cord Injury-Validation of Existing Literature and Relation to Selected Potentially Modifiable Comorbidities: A Prospective Canadian Cohort Study. *J Neurotrauma, 34*(20), 2883-2891. doi:10.1089/neu.2016.4933  Min, L., Ubhayakar, N., Saliba, D., Kelley-Quon, L., Morley, E., Hiatt, J., . . . Tillou, A. (2011). The vulnerable elders survey-13 predicts hospital complications and mortality in older adults with traumatic injury: a pilot study. *J Am Geriatr Soc, 59*(8), 1471-1476. doi:10.1111/j.1532-5415.2011.03493.x  Moulton, L. S., Green, N. L., Sudahar, T., Makwana, N. K., & Whittaker, J. P. (2015). Outcome after conservatively managed intracapsular fractures of the femoral neck. *Ann R Coll Surg Engl, 97*(4), 279-282. doi:10.1308/003588415X14181254788809  Muakkassa, F. F., Marley, R. A., Workman, M. C., & Salvator, A. E. (2010). Hospital outcomes and disposition of trauma patients who are intubated because of combativeness. *J Trauma, 68*(6), 1305-1309. doi:10.1097/TA.0b013e3181dcd137  Muller, M. C., Juptner, U., Wullner, U., Wirz, S., Turler, A., Wirtz, D. C., . . . Standop, J. (2008). [Parkinson's disease influences the perioperative risk profile in trauma patients]. *Z Orthop Unfall, 146*(2), 227-230. doi:10.1055/s-2008-1038330  Muraki, S., Yamamoto, S., Ishibashi, H., & Nakamura, K. (2006). Factors associated with mortality following hip fracture in Japan. *J Bone Miner Metab, 24*(2), 100-104. doi:10.1007/s00774-005-0654-z  Myers, A. H., Palmer, M. H., Engel, B. T., Warrenfeltz, D. J., & Parker, J. A. (1996). Mobility in older patients with hip fractures: examining prefracture status, complications, and outcomes at discharge from the acute-care hospital. *J Orthop Trauma, 10*(2), 99-107. doi:10.1097/00005131-199602000-00005  Norton, L. W., Sweeney, M., & Eiseman, B. (1975). Incidental appendicectomy with laparotomy for trauma. *Br J Surg, 62*(6), 487-489. doi:10.1002/bjs.1800620614  O'Keefe, G. E., Maier, R. V., Diehr, P., Grossman, D., Jurkovich, G. J., & Conrad, D. (1997). The complications of trauma and their associated costs in a level I trauma center. *Arch Surg, 132*(8), 920-924; discussion 925. doi:10.1001/archsurg.1997.01430320122021  Oosthuizen, G. V., Clarke, D. L., Laing, G. L., Bruce, J., Kong, V. Y., Van Staden, N., & Muckart, D. J. (2013). Introducing video-assisted thoracoscopy for trauma into a South African township hospital. *World J Surg, 37*(7), 1652-1655. doi:10.1007/s00268-013-2026-5  Park, C. Y., Choi, H. Y., You, N. K., Roh, T. H., Seo, S. J., & Kim, S. H. (2016). Continuous Renal Replacement Therapy for Acute Renal Failure in Patients with Traumatic Brain Injury. *Korean J Neurotrauma, 12*(2), 89-93. doi:10.13004/kjnt.2016.12.2.89  Pi, H. Y., Gao, Y., Wang, J., Hu, M. M., Nie, D., & Peng, P. P. (2016). Risk Factors for In-Hospital Complications of Fall-Related Fractures among Older Chinese: A Retrospective Study. *Biomed Res Int, 2016*, 8612143. doi:10.1155/2016/8612143  Pillay, J., van der Wouden, J. C., & Leenen, L. P. (2007). [Retrospective application of the performance indicator 'hip fracture: operate within 24 hours' in 217 patients treated at the University Medical Centre Utrecht in 2000-2003: reduction in postoperative pneumonia but not mortality]. *Ned Tijdschr Geneeskd, 151*(17), 967-970. Retrieved from https://www.ncbi.nlm.nih.gov/pubmed/17520850  Poole, G. V., Ward, E. F., Griswold, J. A., Muakkassa, F. F., & Hsu, H. S. (1992). Complications of pelvic fractures from blunt trauma. *Am Surg, 58*(4), 225-231. Retrieved from https://www.ncbi.nlm.nih.gov/pubmed/1586080  Rostagno, C., Buzzi, R., Campanacci, D., Boccacini, A., Cartei, A., Virgili, G., . . . Marchionni, N. (2016). In Hospital and 3-Month Mortality and Functional Recovery Rate in Patients Treated for Hip Fracture by a Multidisciplinary Team. *PLoS One, 11*(7), e0158607. doi:10.1371/journal.pone.0158607  Rothner, O. (1980). [The treatment of medial fractures of the femoral neck (author's transl)]. *Wien Med Wochenschr, 130*(2), 76-78. Retrieved from https://www.ncbi.nlm.nih.gov/pubmed/7385894  Santos, E. A., Santos Filho, W. J., Possatti, L. L., Bittencourt, L. R., Fontoura, E. A., & Botelho, R. V. (2012). Clinical complications in patients with severe cervical spinal trauma: a ten-year prospective study. *Arq Neuropsiquiatr, 70*(7), 524-528. doi:10.1590/s0004-282x2012000700010  Sariego, J., Brown, J. L., Matsumoto, T., & Kerstein, M. D. (1993). Predictors of pulmonary complications in blunt chest trauma. *Int Surg, 78*(4), 320-323. Retrieved from https://www.ncbi.nlm.nih.gov/pubmed/8175260  Sasabuchi, Y., Matsui, H., Lefor, A. K., Fushimi, K., & Yasunaga, H. (2018). Timing of surgery for hip fractures in the elderly: A retrospective cohort study. *Injury, 49*(10), 1848-1854. doi:10.1016/j.injury.2018.07.026  Schulze Raestrup, U., & Smektala, R. (2006). [Are there relevant minimum procedure volumes in trauma and orthopedic surgery?]. *Zentralbl Chir, 131*(6), 483-492. doi:10.1055/s-2006-955451  Sever, R., Gold, A., Segal, O., Regev, G., Keynan, O., Salai, M., & Justo, D. (2012). Admission Norton scale scores (ANSS) are associated with post-operative complications following spine fracture surgery in the elderly. *Arch Gerontol Geriatr, 55*(1), 177-180. doi:10.1016/j.archger.2011.08.007  Starcevic, S., Suljagic, V., Stamenkovic, D., Bokonjic, D., & Munitlak, S. (2016). In-hospital mortality analysis in patients with proximal femoral fracture operatively treated by hip arthroplasty procedure. *Vojnosanit Pregl, 73*(3), 251-255. doi:10.2298/vsp150204088s  Suero, E. M., Meindl, R., Schildhauer, T. A., & Citak, M. (2018). Clinical Prediction Rule for Heterotopic Ossification of the Hip in Patients with Spinal Cord Injury. *Spine (Phila Pa 1976), 43*(22), 1572-1578. doi:10.1097/BRS.0000000000002680  Tornetta, P., 3rd, Mostafavi, H., Riina, J., Turen, C., Reimer, B., Levine, R., . . . Homel, P. (1999). Morbidity and mortality in elderly trauma patients. *J Trauma, 46*(4), 702-706. doi:10.1097/00005373-199904000-00024  Velmahos, G. C., Toutouzas, K., Chan, L., Tillou, A., Rhee, P., Murray, J., & Demetriades, D. (2003). Intubation after cervical spinal cord injury: to be done selectively or routinely? *Am Surg, 69*(10), 891-894. Retrieved from https://www.ncbi.nlm.nih.gov/pubmed/14570369  Wahman, K., Nilsson Wikmar, L., Chlaidze, G., & Joseph, C. (2019). Secondary medical complications after traumatic spinal cord injury in Stockholm, Sweden: Towards developing prevention strategies. *J Rehabil Med, 51*(7), 513-517. doi:10.2340/16501977-2568  Yamauchi, Y., Yasunaga, H., Sakamoto, Y., Hasegawa, W., Takeshima, H., Urushiyama, H., . . . Nagase, T. (2016). Mortality associated with bone fractures in COPD patients. *Int J Chron Obstruct Pulmon Dis, 11*, 2335-2340. doi:10.2147/COPD.S112142  Zellweger, R., Navsaria, P. H., Hess, F., Omoshoro-Jones, J., Kahn, D., & Nicol, A. (2004).  Transdiaphragmatic pleural lavage in penetrating thoracoabdominal trauma. *Br J Surg, 91*(12), 1619-1623. doi:10.1002/bjs.4598 |

| **Supplemental Table 3.** A clarification of used MINORS criteria in this study^16^. | | | |
| --- | --- | --- | --- |
| **Methodological items** | **2**  (MINORS: reported and adequate) | **1**  (MINORS: reported but inadequate) | **0**  (MINORS: not reported) |
| **A clearly stated aim** | Aim and/or hypothesis with outcome(s) was reported in the introduction and/or methods sections. | Aim and/or hypothesis without outcome(s) was reported in the introduction and/or methods sections. | Not reported in the introduction and/or methods section(s). |
| **Inclusion of consecutive patients** | Explicit in- and exclusion criteria and a study period were reported in the methods section. | Either the study period or in- and exclusion criteria were not reported in the methods section. | No reported in- and exclusion criteria and reported study period in the methods section. |
| **Prospective collection of data** | Prospective study (i.e., the data was primarily collected for the stated research question/aim); reported or directly deductible from methods or rationale. | Retrospective; reported or directly deductible from methods or rationale. | Not reported or not deductible from methods or rationale. |
| **Endpoints appropriate to the aim of the study** | Outcomes are appropriate to the aim of the study and stated in the methods section. | - Outcomes are not appropriate to the aim of the study. - Outcomes are appropriate to the aim of the study but not specifically stated in the methods section but deductible from the other sections. | Not reported. |
| **Unbiased assessment of the study endpoint** | Blind evaluation of outcomes. | Blinding was reported but not performed. | Not reported. |
| **Follow-up period appropriate to the aim of the study** | Appropriate follow-up period that is clearly reported in the methods section. | - Inappropratie follow-up period that is clearly reported in the methods section. - Appropriate follow-up period that is not clearly reported (e.g., deductable from methods or results sections). | - Inappropriate and not clearly reported follow-up period. - Not reported. |
| **Loss to follow up less than 5%** | - ≤5% loss to follow-up. - ≤5% missing data. | - >5% and ≤20% loss to follow-up. - >5% and ≤20% missing data with proper imputation technique. - ≤5% missing data without proper imputation technique. | - Loss to follow-up and/or missing data not reported. - More than 20% loss to follow-up. - More than 20% missing data. - >5% and ≤20% missing data without proper imputation technique. |
| **Prospective calculation of the study size** | A sample size calculation or a priori power analysis has been performed and properly reported (i.e., including effect size, significance, and power/sample size). | A sample size calculation has been reported but not properly reported. | - No sample size reported.   A post hoc power analysis was performed. |
| **An adequate control group** | Reported and adequate. | - Reported but inadequate control group. - Not applicable. | Not reported. |
| **Contemporary groups** | Study and control groups have been managed and/or included during the same period of time. | Study and control groups have not been managed and/or included during the same period of time. | Not reported. |
| **Baseline equivalence of groups** | Baseline characteristics of groups have been described clearly and are comparable. | - Baseline characteristics of groups have been described clearly but are not comparable. - Baseline characteristics of groups are comparable but insufficiently described (e.g., little or inappropratie variables). | - Not reported. - Baseline characteristics are not comparable and have been insufficiently described. |
| **Adequate statistical analyses** | Statistical analyses have been described and are appropriate for the aim of the study. | - Statistical analyses have been described but are inappropriate for the study aim. - Statistical analyses are appropriate for the study aim but poorly/partly reported. | - Statistical analyses are inappropriate for the study aim and poorly/partly reported. - Not reported. |

| **Supplemental Table 4.** Used clinical criteria on hospital-acquired pneumonia in trauma patient research. | | |
| --- | --- | --- |
| **Author(s)** | **Year** | **Used criterion/criteria** |
| Seok^19^ | 2019 | Fullfillment of at least 2 of the following criteria:   - Body temperature >38.3°C - Leukocytosis >11,000/dL - Purulent sputum - Aggravation of chest radiograph findings |
| Conradsson^20^ | 2019 | Pneumonia was confirmed:   - Radiographically combined with   - either sputum or   - blood tests |
| Warren^18^ | 2019 | CDC 2018 PNU1 criteria for non-ventilator-associated hospital-acquired pneumonia |
| Wutzler^17^ | 2019 | The presence of new progressive infiltrate accompanied by at least two of the following symptoms:   - Body temperature ≥ 38°C or ≤ 35°C - Leukocytosis (white blood cell count of ≥10,000/mm3) or leukopenia (white blood cell count of ≤4500/mm3, or more than 15% immature neutrophils) - Purulent respiratory secretions |
| Djuric^23^ | 2018 | - CDC 2014 criteria   and/or   - ECDC 2012 criteria |
| Guo^22^ | 2018 | - ATS 2005 criteria   Furthermore:   - Attending physician diagnosis - Confirmed diagnosis by quality improvement committee |
| Yadollahi^21^ | 2018 | CDC/NHSN criteria |
| Denis^24^ |  | Pneumonia was diagnosed using clinical features and confirmed by a radiologist using chest X-rays.  GUIDELINE: Respiratory Management Following Spinal Cord Injury: A Clinical Practice Guideline For Healthcare Professionals (2005) |
| Folbert^26^ | 2017 | Pneumonia was defined as a clinical presentation; the diagnosis was confirmed with   - Imaging, and - Antibiotics were prescribed |
| Yoo^25^ | 2017 | We defined post-operative fever (POF) as any body temperature ≥38°C in the early postoperative period occurring between postoperative day 0 and 14   - The diagnostic workup for POF was deemed to be positive for pneumonia using the following definitions: a positive chest x-ray |
| Curtis^27^ | 2016 | Defined as:   - Radiological evidence of pulmonary air-space opacification, together with - Medical record documentation of a clinical diagnosis of pneumonia and - Treatment with antibiotics   When radiological evidence of pulmonary air-space opacification developed within 24 hours of hospital arrival, these changes were considered to represent contusion rather than infection |
| Ewan^61^ | 2015 | HAP was recorded when antibiotics were started for pneumonia by the responsible clinician after 48 hours in hospital. The diagnosis was further characterised using American Thoracic Society (ATS) and British Society for Antimicrobial Chemotherapy (BSAC) guidelines, both of which required a chest radiograph with new infiltrates.   - ATS 2005 guidelines also require two of:   - Fever >38°C   - Leukocytosis or leukopenia and   - Purulent secretions - BSAC 2008 guidance suggests:   - Purulent tracheal secretions and   - Increased oxygen requirement   - Leukocytosis >10,000 /mm3 or <4,000/mm3, and   - Core temperature >38.3°C |
| Yun^28^ | 2015 | - New or progressive infiltrates seen on radiologic examination and - Evidence of infection   - Documented body temperature >38°C or <36°C, or   - Peripheral white blood cell count ≥12,000 or <4,000 cells/mm^3^), plus   - One of the following:     - New onset purulent sputum     - Change in character of sputum     - Increase in respiratory secretions     - New onset worsening cough     - Dyspnea or tachypnea, rales/bronchial breath sounds     - Worsening gas exchange     - Same organism isolated from both respiratory and blood cultures     - Positive culture from a minimally contaminated lower respiratory tract specimen     - ≥5% bronchoalveolar lavage-obtained cells containing intracellular bacteria on direct microscopy, or     - Laboratory confirmation of infection with an uncommon pathogen - Patients not meeting this a priori definition, as outlined by the CDC/NHSN *(NB: 2004)*, could receive a clinical diagnosis by the treating physician if they met the diagnostic criteria with concurrent directed antimicrobial therapy for more than five days. |
| Kamiya^29^ | 2015 | Pneumonia was defined as:   - Respiratory distress accompanied by - An infiltrating shadow on plain radiogram - Positive sputum cultures and - An elevated white blood cell count (WBC) or C-reactive protein |
| Landeen^30^ | 2014 | Pneumonia status was determined by documentation in medical records, which included:   - Clinic assessments and/or - Objective surveillance definition (i.e., CDC 2008) |
| Yang^12^ | 2014 | Three alternative definitions of pneumonia were assessed:   - Attending diagnosis on or after hospital day 4 (rather than 2) but within 30 days of presentation (to ensure that the cases were hospital-acquired) - Attending diagnosis that also met American Thoracic Society criteria for pneumonia (to minimize subjectivity in the diagnosis): focal infiltrate on chest radiograph, plus two of three clinical features (fever greater than 38°C, leukocytosis or leukopenia, and purulent secretions) - Diagnosis as determined by our hospital’s quality improvement committee. The latter assessment, though based in part on physician diagnoses, was completely independent of this study and blinded from the primary outcome as determined by our study team |
| Mica^32^ | 2013 | In nonintubated patients, pneumonia was diagnosed only by:   - Conventional chest x-ray studies, and - Elevated inflammatory parameters (C-reactive protein [CRP], interleukin 6 [IL-6], and procalcitonin [PCT]) in the patient’s blood. |
| Hyllienmark^33^ | 2013 | Swedish IC registry criteria:   - A new or progressive infiltrate on either plain chest X-ray or computerised tomography of the chest, together with either:   - Body temperature of 38.5°C and C-reactive protein 100 mg/l or alternatively   - Significant growth of a potential airway pathogen from protected brush specimen (PSB) (103 colonies/ml) or broncho-alveolar lavage (BAL) (104 colonies/ml) - For both alternative 1 and 2, the criteria must be met within a time period starting 48 h before and ending 48 h after the appearance of a new or progressive lung infiltrate |
| Schirmer-Mikalsen^31^ | 2013 | Start of symptoms before 48h of respiratory treatment:   - Infiltrates on chest X-ray - Leukocytes > 10 or < 3 x 10^9^/l - Temp. > 38.5°C or < 35°C, and - Purulent tracheal secretions |
| Yeung^34^ | 2012 | The Trauma attending who makes the diagnosis follows the Centers for Disease Control guidelines for diagnosing pneumonia, which include clinical suspicion and new and persistant (>48 h) or progressive radiographic infiltrate plus two of the following: temperature >38°C or <36°C, blood leukocyte count >10,000 cells/mL or <5000 cells/mL, purulent tracheal secretions, and gas exchange degradation.  N.B.: The in-article reference is to the American Thoracic Society 2005 guideline, these were noted in the criteria overview. |
| Hakim^35^ | 2012 | CDC 2008 criteria |
| Strumwasser^36^ | 2011 | CDC 2008 criteria |
| Becher^23^ | 2011 | Hospital-acquired pneumonia was defined as pneumonia that occurred 48h or more after admission. Pneumonia was suspected on clinical grounds:   - Systemic inflammatory response syndrome (SIRS) response - Worsening respiratory function, and - New or progressive infiltrates on chest radiograph - The diagnosis was confirmed with quantitative cultures of lower respiratory secretions; the majority of the cultures were obtained by bronchoalveolar lavage (BAL), with a small number of quantitative deep endotracheal aspirates. Identification of a primary causative pathogen was based on final culture results; a diagnosis of pneumonia was made if a threshold concentration of >10^5 colony-forming units (CFU)/mL was reached. |
| Karunakar^24^ | 2010 | Pneumonia was diagnosed after   - A chest x-ray with one or more lobe infiltrates - White blood cell count greater than 13,000 mL - Fever, and - Increasing oxygen requirement or a bronchoalveolar lavage positive for pathogenic bacteria. |
| Worrall^25^ |  | ATS/IDSA guideline based:   - A new or progressive radiographic infiltrate plus - Two of three clinical features:   - Fever >38°C,   - Leukocytosis (white blood cell count >12,000 K/mm^3^) or   - Leukopenia (white blood cell count <4,000 K/mm^3^), or   - Purulent respiratory secretions. |
| García-Alvarez^26^ | 2010 | CDC 1996 definition |
| Friese^27^ | 2008 | The diagnosis of pneumonia was established by:   - The presence of a new or progressive infiltrate on chest radiograph in conjunction with ≥1 of the following:   - Leukocytosis or leukopenia   - Core temperature >38°C, and   - A change in sputum character |
| Schirmer-Mikalsen^28^ | 2007 | Start of symptoms before 48h of respiratory treatment:   - Infiltrates on chest X-ray - Leukocytes > 10 or < 3 x 10^9^/l - Temp. > 38.5°C or < 35°C, and - Purulent tracheal secretions |
| Giamberardino^29^ | 2007 | CDC 1996 definition |
| Bochicchio^31^ | 2004 | Absolute criteria – all three of these criteria were required for the diagnosis of pneumonia:   - A new or increasing infiltrate on chest film. - Purulent tracheobronchial secretions. - Sputum Gram stain with many polymorphonuclear neutrophils, <10 epithelial cells, and the predominance of one organism.   Additional criteria:   - Fever and temperature >38°C. - Leukocytosis or leukopenia. - Rales or dullness on percussion on chest physical examination. - No improvement on chest film after two or three treatments of chest physiotherapy over a 6-h period. - Respiratory function worsening (i.e., increased ventilator support). |
| Kamel^32^ | 2003 | X-ray criteria |
| McKinley^33^ | 2002 | Clinical and radiological evidence of pneumonia |
| Carson^34^ | 1999 | Pneumonia was defined by:   - Chest radiograph consistent with infiltrate and antibiotic treatment, or - Physician diagnosis of pneumonia and subsequent antibiotic treatment |
| Claxton^35^ | 1998 | - Temperature >38.5C - White blood count > 12.0 x 10^9^ L - Positive sputnm culture and - New infiltrate on chest radiograph |
| Bozorgzadeh^36^ | 1999 | CDC criteria, unknown year. The in-article reference of surgical site infection is to Horan 1999. We therefore assumed that the CDC 1988 criteria for pneumonia diagnosis were used. |
| Gonzalez^37^ | 1998 | Chest radiograph diagnostic for a new or progressive infiltrate, consolidation, or pleural effusion plus any one of the following:   - Purulent sputum - Pathogen isolated from a blood culture; or - Pathogen isolated from a transtracheal aspirate, bronchial brushing, or biopsy specimen |
| Allen^38^ | 1997 | Pneumonia was diagnosed in the presence of:   - A pulmonary infiltrate on chest x-ray and if three of four clinical criteria existed:   - Fever   - Leukocytosis   - Purulent sputum, and   - New or progressive roentgenographic infiltrate - Supported by the identification of a pathogen in either the endotracheal aspirate or bronchoalveolar lavage |
| Morrison^39^ | 1996 | A pulmonary infection that resulted in treatment with antibiotics |
| Renz^40^ | 1995 | CDC 1988 criteria |
| Nichols^41^ | 1994 | CDC 1988 criteria |
| Beraldo^42^ | 1993 | All patients with:   - More than two episodes of fever (axillary temperature > 38°C) for 24 hours before and whilst undergoing treatment - Cough - Yellow sputum - Chest x-rays showing consolidation, and - Positive bacterial sputum culture. |
| Rello^43^ | 1992 | - Presence of a new and persistent lung opacity on the chest x-ray film, excluding those with clinical evidence of noninfectious origin, plus two of the following items:   - Fever >38°C,   - Leukocytosis > 10,000/cu mm,   - Purulent respiratory secretions |
| Moore^44^ | 1989 | Lobar pneumonia included fibrile patients with:   - Roentgenographic changes indicating at least lobar involvement with - A positive sputum culture |
| Moore^45^ | 1989 | Diagnostic criteria for pneumonia included:   - Fever - Leukocytosis - Purulent sputum samples, and - A new infiltratie on chest X-ray studies |
| LoCurto^46^ | 1986 | An inflammatory process involving the pulmonary parenchyma, identified on   - Chest radiogram and accompanied by - Clinical signs and symptoms - Fever, and - Leukocytosis, with - Positive sputum cultures |
| Grover^47^ | 1977 | Radiographic evidence |
| **Abbreviations:** ATS, American Thoracic Society; BSAC, British Society for Antimicrobial Chemotherapy; CDC, Center for Disease Control; ECDC, European CDC; IC, Intensive Care unit; ICD, International Classification of Diseases; IDSA, Infectious Diseases Society of America; NHSN, National Healthcare Safety Network; NNIS, National Nosocomial Infections Surveillance; | | |

| **Supplemental Table 5.** Complete overview of the MINORS criteria scoring of included studies. | | | | | | | | | | | | | |
| --- | --- | --- | --- | --- | --- | --- | --- | --- | --- | --- | --- | --- | --- |
| **First author (year)** | **MINORS criteria**^§^ | | | | | | | | | | | | |
|  | A clearly stated aim | Inclusion of consecutive patients | Prospective collection of data | Endpoints appropriate to the aim of the study | Unbiased assessment of the study endpoint | Follow-up period appropriate to the aim of the study | Loss to follow up less than 5% | Prospective calculation of the study size | An adequate control group | Contemporary groups | Baseline equivalence of groups | Adequate statistical analyses | **Total score** |
| **Seok^19^** | 1 | 2 | 1 | 2 | 0 | 0 | 1 | 0 | 2 | 2 | 1 | 2 | 14 |
| **Conradsson^20^** | 2 | 2 | 2 | 1 | 0 | 1 | 0 | 0 | 2 | 2 | 0 | 2 | 14 |
| **Warren^18^** | 2 | 1 | 1 | 1 | 0 | 0 | 0 | 0 | 2 | 2 | 1 | 2 | 12 |
| **Wutzler^17^** | 2 | 2 | 2 | 1 | 0 | 1 | 0 | 0 | 2 | 2 | 2 | 2 | 15 |
| **Djuric^23^** | 2 | 2 | 2 | 2 | 2 | 2 | 1 | 0 | 2 | 2 | 1 | 2 | 20 |
| **Guo^22^** | 2 | 1 | 2 | 1 | 0 | 2 | 0 | 0 | 2 | 2 | 1 | 2 | 15 |
| **Yadollahi^21^** | 2 | 2 | 2 | 2 | 0 | 0 | 0 | 0 | 2 | 2 | 0 | 0 | 11 |
| **Denis^24^** | 1 | 2 | 2 | 2 | 0 | 2 | 0 | 0 | 2 | 2 | 1 | 2 | 16 |
| **Folbert^26^** | 2 | 2 | 1 | 2 | 0 | 1 | 0 | 0 | 2 | 2 | 1 | 2 | 14 |
| **Yoo^25^** | 2 | 2 | 1 | 2 | 0 | 2 | 0 | 2 | 2 | 1 | 1 | 2 | 17 |
| **Curtis^27^** | 2 | 2 | 2 | 2 | 0 | 2 | 2 | 2 | 2 | 2 | 1 | 2 | 21 |
| **Ewan^61^** | 2 | 2 | 2 | 1 | 0 | 2 | 0 | 0 | NA | NA | NA | NA | 9* |
| **Yun^28^** | 2 | 2 | 1 | 2 | 0 | 2 | 0 | 0 | NA | NA | NA | NA | 9* |
| **Kamiya^29^** | 2 | 2 | 1 | 1 | 0 | 0 | 1 | 0 | 2 | 2 | 1 | 2 | 14 |
| **Landeen^30^** | 2 | 2 | 1 | 2 | 1 | 2 | 0 | 1 | 2 | 2 | 1 | 2 | 18 |
| **Yang^12^** | 2 | 2 | 1 | 1 | 0 | 2 | 0 | 0 | 2 | 2 | 1 | 2 | 15 |
| **Mica^32^** | 2 | 2 | 1 | 2 | 0 | 2 | 0 | 0 | 2 | 2 | 1 | 2 | 16 |
| **Hyllienmark^33^** | 2 | 2 | 2 | 2 | 0 | 2 | 2 | 0 | NA | NA | NA | NA | 12* |
| **Schirmer-Mikalsen^31^** | 2 | 2 | 1 | 2 | 0 | 0 | 0 | 0 | 2 | 2 | 1 | 2 | 14 |
| **Yeung^34^** | 2 | 2 | 2 | 2 | 1 | 2 | 1 | 2 | 2 | 2 | 2 | 2 | 22 |
| **Hakim^35^** | 2 | 2 | 1 | 2 | 0 | 0 | 0 | 0 | NA | NA | NA | NA | 7* |
| **Strumwasser^36^** | 2 | 2 | 1 | 1 | 0 | 1 | 0 | 0 | NA | NA | NA | NA | 7* |
| **Becher^23^** | 2 | 2 | 1 | 1 | 0 | 0 | 0 | 0 | 2 | 2 | 1 | 2 | 12 |
| **Karunakar^24^** | 2 | 2 | 1 | 2 | 0 | 1 | 0 | 1 | 2 | 1 | 2 | 2 | 16 |
| **Worrall^25^** | 2 | 2 | 2 | 1 | 0 | 1 | 0 | 0 | 0 | 0 | 0 | 2 | 10 |
| **García-Alvarez^26^** | 2 | 2 | 1 | 1 | 0 | 0 | 0 | 0 | 2 | 1 | 0 | 2 | 11 |
| **Friese^27^** | 2 | 2 | 1 | 0 | 1 | 2 | 1 | 0 | 0 | 2 | 0 | 2 | 13 |
| **Schirmer-Mikalsen^28^** | 1 | 2 | 1 | 1 | 0 | 1 | 0 | 0 | 1 | 2 | 1 | 2 | 11 |
| **Giamberardino^29^** | 2 | 2 | 1 | 1 | 0 | 1 | 0 | 0 | 0 | 2 | 0 | 2 | 9 |
| **Bochicchio^31^** | 2 | 2 | 2 | 1 | 0 | 0 | 0 | 0 | 0 | 2 | 0 | 2 | 11 |
| **Kamel^32^** | 1 | 2 | 1 | 1 | 0 | 1 | 0 | 0 | 0 | 2 | 0 | 2 | 14 |
| **McKinley^33^** | 2 | 1 | 2 | 2 | 0 | 2 | 0 | 0 | 1 | 2 | 0 | 2 | 12 |
| **Carson^34^** | 2 | 2 | 1 | 2 | 0 | 0 | 0 | 0 | 1 | 2 | 0 | 2 | 12 |
| **Claxton^35^** | 2 | 2 | 1 | 2 | 0 | 1 | 0 | 0 | 2 | 2 | 1 | 2 | 14 |
| **Bozorgzadeh^36^** | 2 | 1 | 2 | 1 | 0 | 0 | 0 | 0 | 2 | 2 | 1 | 2 | 15 |
| **Gonzalez^37^** | 2 | 2 | 2 | 2 | 2 | 0 | 0 | 0 | 2 | 2 | 0 | 1 | 15 |
| **Allen^38^** | 1 | 1 | 1 | 1 | 0 | 0 | 0 | 0 | 1 | 2 | 0 | 1 | 8 |
| **Morrison^39^** | 2 | 2 | 1 | 1 | 0 | 1 | 0 | 0 | NA | NA | NA | NA | 6* |
| **Renz^40^** | 2 | 2 | 2 | 1 | 0 | 2 | 0 | 0 | NA | NA | NA | NA | 8* |
| **Nichols^41^** | 2 | 2 | 2 | 1 | 2 | 2 | 0 | 2 | 2 | 2 | 0 | 2 | 19 |
| **Beraldo^42^** | 1 | 1 | 0 | 1 | 0 | 1 | 0 | 0 | NA | NA | NA | NA | 4* |
| **Rello^43^** | 2 | 2 | 2 | 1 | 0 | 2 | 0 | 0 | 2 | 2 | 0 | 2 | 14 |
| **Moore^44^** | 1 | 2 | 2 | 2 | 0 | 0 | 0 | 0 | 2 | 2 | 2 | 2 | 15 |
| **Moore^45^** | 0 | 1 | 2 | 1 | 0 | 0 | 1 | 0 | 2 | 2 | 2 | 2 | 13 |
| **LoCurto^46^** | 2 | 2 | 2 | 1 | 0 | 0 | 0 | 0 | 2 | 2 | 1 | 1 | 13 |
| **Grover^47^** | 1 | 1 | 2 | 1 | 2 | 0 | 0 | 0 | 2 | 2 | 0 | 1 | 13 |
| ^§^The study quality was measured using the MINORS criteria; the potential score ranges from 0 (lowest) to 16 or 24 (highest).  *These studies could score a maximum of 16 points, as they were non-comparative studies. | | | | | | | | | | | | | |
